# Peripheral Inflammation Is Associated With Greater Neuronal Injury and Lower Episodic Memory Among Late Middle‐Aged Adults

**DOI:** 10.1111/jnc.70222

**Published:** 2025-09-08

**Authors:** Mona‐Lisa Malarte, Konstantinos Chiotis, Konstantinos Ioannou, Elena Rodriguez‐Vieitez

**Affiliations:** ^1^ Division of Clinical Geriatrics, Center for Alzheimer Research, Department of Neurobiology, Care Sciences and Society Karolinska Institutet Stockholm Sweden; ^2^ Department of Neurology Karolinska University Hospital Stockholm Sweden; ^3^ Division of Neurogeriatrics, Center for Alzheimer Research, Department of Neurobiology, Care Sciences and Society Karolinska Institutet Stockholm Sweden

**Keywords:** ^18^F‐MK6240 tau PET, episodic memory, neuronal injury, peripheral inflammation, plasma NfL, preclinical Alzheimer's disease

## Abstract

Elucidating the earliest biological mechanisms underlying Alzheimer's disease (AD) is critical for advancing early detection strategies. While amyloid‐β (Aβ) and tau pathologies have been central to preclinical AD research, the roles of peripheral biological processes in disease initiation remain underexplored. We investigated patterns of ^18^F‐MK6240 tau positron emission tomography (PET) and peripheral inflammation across stages defined by Aβ burden and neuronal injury in *n* = 132 (64.5 ± 3.4 years old, 69.7% female, 10.7 ± 4.0 years of education, 34.1% APOE4 carriers) cognitively unimpaired late middle‐aged Hispanic adults. ^18^F‐MK6240 tau PET imaging revealed early entorhinal and neocortical tau deposition even in individuals lacking biomarker evidence of neuronal injury as measured by plasma neurofilament light (NfL). Peripheral inflammatory markers were not directly associated with Aβ or tau load but exhibited robust associations with neuronal injury (plasma NfL). Importantly, the hallmark biomarkers of AD proteinopathy (Aβ and tau) did not show a significant association with episodic memory performance, whereas peripheral inflammation and plasma NfL markers demonstrated links to subtle episodic memory impairment. Furthermore, Aβ and tau deposition appeared primarily influenced by genetic predisposition and sex, whereas peripheral inflammation was strongly associated with both neuronal injury (plasma NfL) and comorbidities including higher Body Mass Index (BMI) and Diabetes Mellitus (DM). These findings reveal a complex interplay between central and peripheral mechanisms in the potential earliest phases of AD pathophysiology and argue for the integration of peripheral inflammatory and neurodegeneration markers into models of preclinical AD progression. Recognizing the heterogeneity of early biological changes could refine risk stratification, biomarker development, and preventative strategies targeting inflammation and vascular health in cognitively unimpaired individuals at risk for AD.

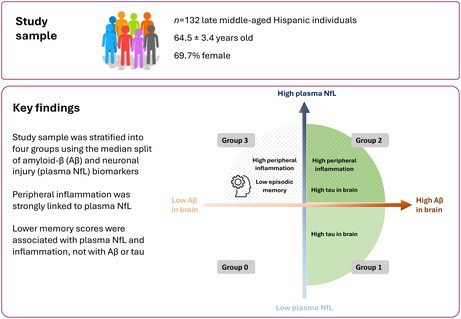

## Graphical abstract

While amyloid‐β and tau are considered core biomarkers of Alzheimer's disease (AD), the role of peripheral biological processes is poorly understood. We investigated peripheral inflammatory markers, tau PET imaging, and episodic memory in cognitively unimpaired Hispanic individuals, stratified into four groups defined by dichotomous amyloid‐β and neuronal injury biomarkers. Our study revealed that peripheral inflammation was associated with greater neuronal injury and lower episodic memory in Hispanic individuals, a group that has been largely underrepresented in AD research. Recognizing the heterogeneity of early biological changes could refine risk stratification, biomarker development, and preventative strategies in individuals at risk for AD.

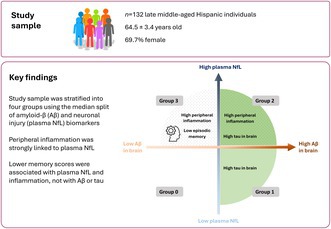

**Abbreviations**
AD: Alzheimer's disease
ADNI: Alzheimer's disease neuroimaging initiative
APOE: apolipoprotein E
Aβ: amyloid‐β
BMI: Body Mass Index
CI: 95% confidence interval
CT: computed tomography
CTh: cortical thickness
CU: cognitively unimpaired
CV: coefficient of variation
CVD: cerebrovascular diseases
CVI: cerebrovascular injury
DM: Diabetes Mellitus
FLAIR: fluid attenuated inversion recovery
LOD: limit of detection
MCI: mild cognitive impairment
MRI: magnetic resonance imaging
NfL: neurofilament light
NPX: Normalized Protein eXpression
PET: positron emission tomography
QC: quality control
QQ plot: quantile‐quantile plot
ROI: region of interest
RRID: Research Resource Identifier (see scicrunch.org)
SD: standard deviation
SRT: Selective Reminding Test
SUV: standardized uptake value
SUVR: standardized uptake value ratio
WMH: white matter hyperintensities

## 1 Introduction

Alzheimer's disease (AD) is increasingly prevalent due to an aging global population (Skaria 2022). AD neuropathological changes typically begin in middle adulthood, decades before clinical symptoms (Beason‐Held et al. 2013; Lloret et al. 2021). Early detection remains challenging as cognitive functions decline with age and with other pathologies such as cerebrovascular diseases (CVD) (Murman 2015; Verdelho et al. 2021). Understanding the mechanisms by which CVD and neurodegeneration mutually influence each other, and how they contribute to or exacerbate cognitive decline, remains crucial, especially in differentiating aging‐related cognitive changes from early neurological conditions.

Given the potential efficacy of new drugs in the early stages of AD (Huang et al. 2023), early diagnosis becomes imperative, emphasizing the need for a deeper understanding of how AD interacts with other conditions before implementing biomarkers in clinical practice. Diagnostic markers for CVD, such as strokes and white matter hyperintensities (WMH) (Guo and Shi 2022) are commonly observed in the brains of AD patients (Duara and Barker 2022). Risk factors like Diabetes Mellitus (DM) and high Body Mass Index (BMI) are linked to cognitive impairment (Balasubramanian et al. 2021; Ehtewish et al. 2022), underlining the significance of discerning how these factors individually or collectively influence cognitive decline.

In this context, refining biomarker‐based stratification of potential preclinical AD, especially in diverse and high‐risk populations is increasingly important. Despite a growing body of research, Hispanic individuals remain underrepresented in biomarker studies (Acosta et al. 2020). The prevalence of dementia is rapidly escalating within this community, with AD affecting about 12% of the elderly Latino population, the highest rate of increase observed among any ethnic group (Llibre Rodriguez et al. 2008; Nitrini et al. 2009; Prina et al. 2019; Rocca et al. 2011). Hispanics in the Southwestern US are diagnosed with dementia earlier than white non‐Hispanics (Fitten et al. 2014), highlighting the need for inclusive research that accounts for ethnic and racial diversity in disease presentation and progression.

Recent efforts in AD research have focused on identifying biomarkers in the preclinical phase, conceptualizing AD as a systemic disease that affects both central and peripheral immune systems (Bettcher et al. 2021). Identifying early dementia risk markers requires examining proteins and brain networks showing abnormal patterns in midlife adults and understanding peripheral biological pathways from the early stages of neurodegenerative diseases. The ATN classification system incorporates biomarkers of amyloid‐β (Aβ) and tau pathologies along with non‐specific markers of neurodegeneration and neuronal injury such as neurofilament light (NfL) polypeptide (Jack, Bennett, et al. 2016). The updated ATN‐IVS framework incorporates inflammation (I), vascular (V) and α‐synuclein (S) markers in addition to the core ATN system (Hampel et al. 2021; Jack et al. 2024). The variability of plasma NfL across cognitive decline stages (Mielke et al. 2019) suggests diverse clinical presentations and potential comorbidities (Ashton et al. 2021; Narayanan et al. 2021). In particular, inflammation is now recognized as a crucial factor in AD pathogenesis, especially in early stages (Bellaver et al. 2021; Rodriguez‐Vieitez et al. 2016), with recent proposals to include it as an “I” biomarker in the classification (Imbimbo et al. 2023).

Normal aging predisposes to systemic inflammation (“inflammaging”), which may contribute to AD pathophysiology (Leng and Edison 2021). Disturbances in these inflammatory mechanisms, mediated by astrocytes/microglia in the brain, and by macrophages/monocytes in the periphery (Spiteri et al. 2022) are gaining attention. Recent findings indicate that elevated plasma glial fibrillary acidic protein predicts tau pathology and subsequent cognitive impairment in Aβ‐positive individuals (Bellaver et al. 2023). Inflammatory markers in preclinical AD (Engelhart et al. 2004; Walker et al. 2023) differentiate mild cognitive impairment (MCI) progressors and non‐progressors to AD dementia (Kivisäkk et al. 2022). The specific role of inflammation in the pathogenesis of AD could consist of either promoting or delaying disease progression through the dual pro‐ and anti‐inflammatory states of microglia/astrocytes and their secreted cytokines and chemokines (Hickman et al. 2018; Kumar et al. 2023; Sinyor et al. 2020). New techniques enable plasma inflammation measurement, but its interplay with tau burden in the brain, other imaging and plasma biomarkers, and cognition remains poorly understood.

The MK6240 tau tracer detects early‐stage tau deposits in the AD continuum with low non‐specific binding (Betthauser et al. 2020; Kreisl et al. 2022). Higher MK6240 binding was observed in early‐onset compared with late‐onset AD as measured in post‐mortem brain tissues (Malarte et al. 2021), and the MK6240 tracer showed enhanced sensitivity over previously developed tau tracers (Gogola et al. 2022). Our current study extends beyond previous research on MK6240 by investigating it in conjunction with a range of biomarkers including peripheral inflammation, neuronal injury, and episodic memory, offering a comprehensive insight into early AD.

This study aimed to investigate in vivo tau positron emission tomography (PET) and peripheral inflammatory biomarkers in a cohort of late middle‐aged Hispanic adults, specifically: (i) characterizing the pattern of ^18^F‐MK6240 tau burden at different early stages of Aβ pathology and neuronal injury; (ii) investigating the patterns of peripheral inflammatory biomarkers across the same stages; and (iii) exploring the potential associations between tau PET, peripheral inflammation, and episodic memory performance.

## 2 Methods

### 2.1 Study Design and Participants

When planning this study, we found few previous reports on AD biomarkers in diverse populations, particularly in the Hispanic population. To address this research gap, we used cross‐sectional data from the Interdisciplinary Research to Understand the Interplay of Diabetes and Alzheimer's Disease (DiCAD) study at Columbia University, obtained from the AD Knowledge Center (DICAD_StudyDetails). The study sample consisted of *n* = 132 late middle‐aged Hispanic individuals (64.5 ± 3.4 years old, 69.7% female, 10.7 ± 4.0 years of education, 34.1% APOE4 carriers) from Northern Manhattan without cognitive impairment. The participants underwent questionnaires of general health, neuropsychological testing, a multi‐modal magnetic resonance imaging (MRI)‐PET neuroimaging protocol, and plasma sampling. All biomarker data were obtained as part of the DiCAD study. The current analyses, including group stratification and all statistical modeling, were independently performed by the authors, who did not have any contact with the study participants. Thus, the study is blind since the authors are different people than the experimenters.

For the aims of this study, we selected cognitively unimpaired (CU) individuals from the DiCAD cohort, with concurrent data on T1‐weighted MRI, T2‐weighted fluid attenuated inversion recovery (FLAIR) MRI, Aβ PET (^18^F‐florbetaben), tau PET (^18^F‐MK6240), plasma measures of inflammation (Olink inflammation panel) and neuronal injury, and neuropsychological assessments. Since we did not find any previous study reporting on the associations between Olink plasma inflammatory markers and Aβ PET or tau PET, we could not perform a formal statistical power analysis to determine the sample size of our study, and we selected all available CU individuals from DiCAD that had concurrent data on these biomarkers (*n* = 132, Table 1). As an inclusion criterion, the time interval between the tau PET scan and the plasma biomarker sampling had to be less than one year. The time intervals between the image acquisition dates and plasma sampling were: 25.0 ± 49.5 (mean ± standard deviation [SD]) days between MRI and plasma sampling, 13.2 ± 25.7 days between Aβ PET and plasma sampling, and 117.7 ± 97.3 days between tau PET and plasma sampling. The neuropsychological assessments were performed with a time interval of 5.1 ± 10.8 days from the plasma sampling. Demographics (age, sex, years of education, APOE genotyping), BMI, DM, global Aβ PET (Rippon et al. 2022), regional tau PET burden, plasma NfL, and cognitive performance for the study sample (*n* = 132) are shown in Table 1. Using the median split to dichotomize the continuous global Aβ and NfL measures, the study sample was further stratified into four groups: “Aβ‐low, NfL‐low” (Group 0); “Aβ‐high, NfL‐low” (Group 1); “Aβ‐high, NfL‐high” (Group 2); “Aβ‐low, NfL‐high” (Group 3).

**TABLE 1:** Characteristics of the study sample.

	Aβ‐low, NfL‐low (*n* = 32)	Aβ‐high, NfL‐low (*n* = 34)	Aβ‐high, NfL‐high (*n* = 32)	Aβ‐low, NfL‐high (*n* = 34)	Total sample (*n* = 132)
**Age, years**					
Mean (SD)	63.5 (3.4)	64.6 (3.5)	65.0 (3.2)	65.0 (3.4)	64.5 (3.4)
**Sex**					
Male	16 (50.0%)	3 (8.8%)	5 (15.6%)	16 (47.1%)	40 (30.3%)
Female	16 (50.0%)	31 (91.2%)	27 (84.4%)	18 (52.9%)	92 (69.7%)
**Education, years**					
Mean (SD)	10.3 (3.7)	10.7 (4.3)	11.7 (4.2)	10.2 (3.8)	10.7 (4.0)
**APOE4 status**					
Carrier	7 (21.9%)	11 (32.4%)	14 (43.8%)	13 (38.2%)	45 (34.1%)
Non‐carrier	25 (78.1%)	23 (67.6%)	18 (56.3%)	21 (61.8%)	87 (65.9%)
**Body mass index (BMI)**					
Mean (SD)	29.9 (2.9)	29.1 (4.5)	28.0 (4.1)	27.9 (3.6)	28.7 (3.9)
Missing	1 (3.1%)	0 (0%)	1 (3.1%)	0 (0%)	2 (1.5%)
**Diabetes Mellitus (DM)**					
Yes	11 (34.4%)	8 (23.5%)	7 (21.9%)	9 (26.5%)	35 (26.5%)
No	21 (65.6%)	26 (76.5%)	25 (78.1%)	25 (73.5%)	97 (73.5%)
**Global amyloid‐β (Aβ) PET, SUVR**					
Mean (SD)	1.08 (0.03)	1.21 (0.14)	1.26 (0.20)	1.08 (0.04)	1.16 (0.14)
**Entorhinal tau PET, SUVR**					
Mean (SD)	1.11 (0.17)	1.20 (0.28)	1.45 (0.59)	1.15 (0.31)	1.22 (0.39)
**Middle/inferior temporal tau PET, SUVR**					
Mean (SD)	1.17 (0.16)	1.24 (0.15)	1.25 (0.18)	1.16 (0.13)	1.20 (0.16)
**Plasma NfL concentration, pg/mL**					
Mean (SD)	7.54 (2.05)	8.41 (1.64)	16.8 (6.04)	16.8 (8.41)	12.4 (6.91)
**Episodic memory, SRT immediate recall**					
Mean (SD)	39.4 (9.2)	39.8 (10.2)	41.5 (8.1)	34.3 (7.7)	38.7 (9.2)
**Episodic memory, SRT delayed recall**					
Mean (SD)	27.8 (11.6)	27.8 (12.3)	29.2 (12.6)	19.3 (9.1)	26.0 (12.0)

Abbreviations: NfL, neurofilament light; SD, standard deviation; SRT, selective reminding test; SUVR, standardized uptake value ratio.

This study was conducted using publicly available data, requested via the AD Knowledge Portal, managed by the US National Institute of Aging's Alzheimer's Disease Translational Research Program, https://adknowledgeportal.synapse.org, under a signed Controlled‐Access Data Use Certificate to comply with ethical guidelines for the protection of human subjects. All data collection had been previously approved by the Institutional Review Board and the Joint Radiation Safety Commission at Columbia University Irving Medical Center (CUIMC) in New York City, USA. All study participants provided written informed consent. The study was conducted in accordance with the ethical standards of the institutional and national research committee and with the 1964 Helsinki Declaration and its later amendments or comparable ethical standards.

### 2.2 Magnetic Resonance Imaging (MRI)

Brain MRI scans were conducted on a General Electric Signa Premier 3T scanner. Structural T1‐weighted MPRAGE (magnetization‐prepared rapid acquisition gradient echo) MRI images were processed using FreeSurfer to obtain cortical thickness (CTh) measures within regions of interest (ROIs) defined by the Desikan‐Killiany atlas (Fischl et al. 2004). For this study, we selected a global measure of CTh that has been previously used to characterize neurodegenerative changes typical of AD and called the AD‐signature CTh measure (Dickerson et al. 2009). Using T2‐weighted FLAIR MRI, the total volume of white matter hyperintensity (WMH) was quantified and used as a measure of cerebrovascular injury (CVI) (Brickman et al. 2015). WMH volumes were calculated by identifying FLAIR voxels that are 2 standard deviations above the mean using a Gaussian distribution, then summing the adjusted voxel counts for total and regional (frontal, temporal, parietal, occipital lobes) volumes using a standardized atlas for spatial normalization (Brickman et al. 2011, 2012, 2015).

### 2.3 Positron Emission Tomography (PET)

All PET scans were processed at the Imaging Lab at Columbia University Medical Center (CUMC) for quality control (QC) and pre‐processing. QC steps involved visual inspection, motion assessment, protocol adherence, image quality metrics, and data form comparison, as previously described (Rippon et al. 2022). PET methodologies derive from the Alzheimer's Disease Neuroimaging Initiative (ADNI).

#### 2.3.1 Aβ PET Acquisition and Image Processing

Aβ PET scans were performed with the ^18^F‐florbetaben ligand. The injected dose of ^18^F‐florbetaben was 300 (±20%) MBq, using a maximum of 30 μg mass dose, administered as a single slow intravenous bolus (Tahmi et al. 2019). Images were acquired during 20 min starting 90 min after injection. Dynamic PET frames were aligned and averaged to get a static PET image. For each participant, the static PET image was registered with a computed tomography (CT) scan that had been acquired for attenuation correction during PET imaging reconstruction to generate a PET/CT fused image (Rippon et al. 2022). Then, the structural T1‐weighted MRI image in FreeSurfer space of the same individual was registered to the respective PET/CT fused image, and this procedure was used to transfer regional masks and the cerebellar gray matter from FreeSurfer space to the PET image. The standardized uptake value (SUV) was calculated for selected regions and normalized to the cerebellar gray matter used as the reference region to derive SUV ratio (SUVR). A global measure of Aβ burden was extracted and expressed in SUVR units, as previously described (Rippon et al. 2022).

#### 2.3.2 Tau PET Acquisition and Image Processing

Tau PET was conducted with the tau ligand ^18^F‐MK6240. The ^18^F‐MK6240 injected activity was 185 (±20%) MBq, and images were acquired during the 80 to 100 min interval after tracer injection. Dynamic frames were aligned and averaged to get a static tau PET image. This image was co‐registered with the static Aβ PET image. The same FreeSurfer‐derived ROIs from the Aβ PET image processing were applied to the tau PET image. ^18^F‐MK6240 SUVRs were calculated using a modified cerebellar gray matter reference region, consisting of posterior cerebellum and ventral temporal/occipital cortex (Buckner et al. 2004). Individual tau burden was extracted from the bilateral entorhinal and middle/inferior temporal cortices. Entorhinal tau was used as a proxy for aging and early tau deposition in preclinical AD, while middle/inferior temporal tau was used as a proxy for AD‐related neocortical tau (Johnson et al. 2016).

### 2.4 Plasma Biomarkers of Neuronal Injury

For this study, we used two plasma biomarkers of neuronal injury: NfL and total‐tau. Both biomarkers were assessed using highly sensitive single‐molecule array (Simoa) assays by Quanterix (Wilson et al. 2016). The NfL assay had a detection limit of 0.97 pg/mL, and a coefficient of variation (CV) of 4.3%. The total‐tau assay captures all tau isoforms, with a lower detection limit of 0.02 pg/mL; reproducibility and repeatability CVs are 8.5% and 7.7% respectively.

### 2.5 Plasma Inflammatory Proteomics

Inflammatory proteins in plasma were quantified using the Olink inflammation panel, which simultaneously analyzes 92 markers in 1 μL of sample using the Proximity Extension Assay (PEA) method, as previously described (Assarsson et al. 2014). Data underwent QC and normalization, with the final read‐out given in Normalized Protein eXpression (NPX) values on a log2 scale. Higher NPX values indicate higher protein expression. Detailed assay validation can be found on the manufacturer's website (www.olink.com), Olink Bioscience (RRID:SCR_003899). For this study, 18 proteins were excluded from the analysis, for which more than 15% of participants had values below each protein's limit of detection (LOD). A final set of 74 proteins was used in the data analyses.

### 2.6 Neuropsychological Testing

As sensitive measures of episodic memory, we used two verbal learning tests measured using both the immediate and delayed recall versions of the Buschke Selective Reminding Test (SRT) (Tahmi et al. 2021).

### 2.7 Statistical Analyses

Cross‐sectional associations among variables were tested using general linear models. The variables in our study included neuroimaging and plasma biomarkers, demographics, and clinical data. Neuroimaging biomarkers included continuous variables for global Aβ PET, entorhinal and middle/inferior temporal tau PET, AD‐signature CTh, and WMH. Plasma biomarkers included the concentrations of 74 inflammatory proteins, as well as NfL and total‐tau. Demographic variables consisted of age in years, sex (1 = male, 0 = female), years of education, and APOE4 status (1 = ε4 carrier, 0 = ε4 non‐carrier). Clinical data included BMI as a continuous variable and DM as a categorical variable (1 = yes, 0 = no). Neuropsychological data included two measures of episodic memory: SRT immediate recall and SRT delayed recall.

Prior to running general linear models, total WMH values were adjusted for total intracranial volume by taking the ratio. Subsequently, WMH and plasma NfL and total‐tau were log‐transformed using the natural logarithm to improve normality of their distributions. In addition, all continuous variables were z‐transformed via mean centering and unit variance scaling. Subsequently, quantile‐quantile (QQ) plots were used to visually assess the normality of all continuous variables, by comparing their quantiles to the quantiles of a standard normal distribution, and we observed that all QQ plots roughly followed straight diagonal lines and that no outliers were apparent. All general linear models involving cognition were performed, adjusted for age, sex, and education. Results from the general linear models are presented as standardized β coefficients and corresponding 95% confidence intervals (CI). Two‐sided *p*‐values below 0.05 were considered significant. Corrections for multiple inflammatory markers were performed using a false discovery rate (*q* < 0.05) approach, two‐sided tests. All statistical analyses and illustrations were performed in R Studio version 2023.03.386 using R version 4.2.3.

## 3 Results

### 3.1 Initial Analysis of Biomarker‐Demographic Associations

As a first step in the study, we explored the pair‐wise associations between each of the individual biomarkers and each of the demographics and clinical variables to have a better understanding of the underlying patterns in the data.

#### 3.1.1 Age, Sex and APOE4 Effects on Biomarkers

Across the whole cohort (*n* = 132), age was positively associated with lower AD‐signature CTh and higher WMH, but it was not significantly associated with Aβ or tau PET variables, nor with any of the plasma biomarkers. Females had significantly greater global Aβ and middle/inferior temporal tau than males, and higher AD‐signature CTh than males. There were no sex differences in any other imaging or fluid biomarkers. APOE4 carriers had significantly greater global Aβ and entorhinal tau than non‐carriers. APOE4 was not significantly associated with any other imaging or plasma biomarkers in this study. To characterize more in detail the associations between Aβ and tau PET with age, sex, and APOE4 status, we performed multivariable models as follows:

(1) $$\text{Global}\;\mathrm{A\beta}\sim \beta_{0}+\beta_{1}\;\text{APOE4 carrier}+\beta_{2}\;\mathrm{age}+\beta_{3}\;\mathrm{sex}$$

(2a) $$\text{Entorhinal}\;\mathrm{tau}\sim \beta_{0}+\beta_{1}\;\text{APOE4 carrier}+\beta_{2}\;\mathrm{age}+\beta_{3}\;\mathrm{sex}$$

(2b) $$\begin{array}{c} \text{Entorhinal}\;\mathrm{tau}\sim \beta_{0}+\beta_{1}\;\text{Global}\;\mathrm{A\beta}+\beta_{2}\;\text{APOE4 carrier}\\ +\beta_{3}\;\mathrm{age}+\beta_{4}\;\mathrm{sex} \end{array}$$

(3a) $$\begin{array}{c} \text{Middle}/\text{Inferior temporal}\;\mathrm{tau}\sim \beta_{0}+\beta_{1}\;\text{APOE4 carrier}\\ +\beta_{2}\;\mathrm{age}+\beta_{3}\;\mathrm{sex} \end{array}$$

(3b) $$\begin{array}{c} \text{Middle}/\text{Inferior temporal}\;\mathrm{tau}\sim \beta_{0}+\beta_{1}\;\text{Global}\;\mathrm{A\beta}\\ +\beta_{2}\;\text{APOE4 carrier}+\beta_{3}\;\mathrm{age}+\beta_{4}\;\mathrm{sex} \end{array}$$

Greater global Aβ was positively associated with APOE4 carrier status (std. β [95% CI] = 0.497 [0.156 to 0.838], *p* = 0.005) and being female (std. β [95% CI] = 0.558 [0.205 to 0.911], *p* = 0.002) (Equation 1). Entorhinal tau was positively associated with APOE4 status (std. β [95% CI] = 0.432 [0.079 to 0.785], *p* = 0.018) (Equation 2a). When global Aβ was added as an independent predictor (Equation 2b), entorhinal tau was no longer associated with APOE4 status, but it was positively associated with global Aβ (std. β [95% CI] = 0.528 [0.373 to 0.683], *p* = 6.7 × 10^−10^).

Middle/inferior temporal tau was positively associated with being female (std. β [95% CI] = 0.818 [0.471 to 1.165], *p* = 9.5 × 10^−6^) (Equation 3a). When global Aβ was added as an independent predictor (Equation 3b), middle/inferior temporal tau was both positively associated with being female (std. β [95% CI] = 0.668 [0.319 to 1.017], *p* = 0.0003) and with global Aβ (std. β [95% CI] = 0.269 [0.104 to 0.434], *p* = 0.002), but it was no longer associated with APOE4 status.

### 3.2 Clinical Data and Biomarkers

Greater BMI was positively associated with higher inflammation (CCL19, CDCP1, CSF‐1, HGF, IL‐18R1, IL6, MCP‐4, OPG, TNFSF14; all *q* < 0.05, corrected for multiple comparisons, with age and sex included as covariates). DM was associated with higher inflammation (CCL19, CDCP1, Flt3L, HGF, IL‐18R1, LIF‐R, OPG, SLAMF1; all *q* < 0.05, corrected for multiple comparisons, with age and sex included as covariates). BMI or DM were not associated with any other imaging or fluid biomarkers in the study.

### 3.3 ^18^F‐MK6240 Tau PET, Plasma Inflammatory Markers and Episodic Memory

Next, we present the results structured into the three primary aims of the study, to investigate: (i) patterns of ^18^F‐MK6240 tau PET burden at different stages of Aβ pathology and neuronal injury; (ii) patterns of plasma inflammatory biomarkers across the same stages; and (iii) associations between tau PET, plasma inflammation, and episodic memory performance.

#### 3.3.1 Patterns of ^18^F‐MK6240 Tau PET Burden at Different Stages of Aβ Pathology and Neuronal Injury

We dichotomized Aβ and neuronal injury measures using the median split, an approach previously used in other studies (Gottesman et al. 2017; Tahmi et al. 2021). Using this method, we stratified the study sample (*n* = 132) into four groups: “Aβ‐low, NfL‐low” (Group 0); “Aβ‐high, NfL‐low” (Group 1); “Aβ‐high, NfL‐high” (Group 2); “Aβ‐low, NfL‐high” (Group 3) (Table 1).

**FIGURE 1:**
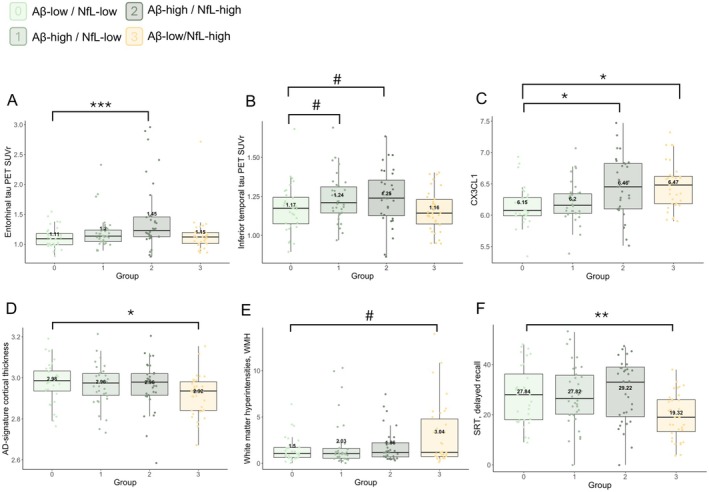
Patterns of neuroimaging, plasma biomarkers and episodic memory, stratified into groups defined by global Aβ and plasma NfL biomarkers. Groups were defined by median‐split dichotomization of global Aβ PET and plasma NfL levels: Group 0 = Aβ‐low/NfL‐low; Group 1 = Aβ‐high/NfL‐low; Group 2 = Aβ‐high/NfL‐high; Group 3 = Aβ‐low/NfL‐high. Significant findings are indicated with symbols: *p < 0.05, **p < 0.01, ***p < 0.001. Statistical trend was denoted by: ^#^ *p* < 0.1.

The patterns of regional tau PET burden across groups are illustrated in Figure 1A,B. Compared with Group 0, Group 1 had a trend‐level higher middle/inferior temporal tau (std. β [95% CI] = 0.405 [−0.067 to 0.877], *p* = 0.096). Compared with Group 0, Group 2 had higher entorhinal tau (std. β [95% CI] = 0.879 [0.413 to 1.345], *p* = 0.0003) and trend‐level higher middle/inferior temporal tau (std. β [95% CI] = 0.465 [−0.015 to 0.945], *p* = 0.060). Group 3 did not show any increased regional tau burden compared with Group 0.

#### 3.3.2 Pattern of Plasma Inflammatory Biomarkers at Different Stages of Aβ Pathology and Neuronal Injury

Compared to Group 0, Group 1 did not show any difference in the concentrations of plasma inflammatory proteins. Group 2 had higher levels of a number of inflammatory proteins (CCL25, CD244, CX3CL1, FGF‐19, IL‐15RA, IL8, MCP‐2, VEGFA; all *p* < 0.05, uncorrected), and lower levels of TRANCE with respect to Group 0 (*p* < 0.05, uncorrected). Group 3 also had higher levels of inflammatory proteins (CCL25, CX3CL1, GDNF; all *p* < 0.05, uncorrected) and lower levels of TNFSF14, TRANCE (*p* < 0.05, uncorrected). The patterns of plasma inflammation across the four groups are illustrated in Figure 1C for a representative plasma protein: CX3CL1.

Complementary analyses across groups were performed for AD‐signature CTh, WMH, and plasma t‐tau. Compared with Group 0, Group 3 had significantly lower AD‐signature CTh (std. β [95% CI] = −0.558 [−1.036 to −0.080], *p* = 0.024) (Figure 1D), and a trend‐level increase in WMH (std. β [95% CI] = 0.448 [−0.034 to 0.930], *p* = 0.070) (Figure 1E), while plasma total‐tau did not show any significant differences across groups (data not shown).

#### 3.3.3 Associations Between Plasma Inflammatory Proteins and Other Biomarkers Across the Whole Study Sample

We performed additional statistical analyses to better understand the association between each of the novel plasma inflammatory proteins and each of the other biomarkers taken individually. From these analyses, we found that a number of plasma inflammatory markers were significantly associated with plasma NfL (that survived correction for multiple comparisons). None of the plasma inflammatory proteins were significantly associated with any other neuroimaging or plasma biomarkers (data not shown). Finally, we built a multivariable general linear model using each plasma inflammatory marker as the dependent variable. Independent predictors included NfL, BMI, and DM (age and sex also included as covariates), as follows:

(4) $$\begin{array}{c} \text{Plasma inflammatory marker}\sim \beta_{0}+\beta_{1}\;\text{Plasma}\;\mathrm{NfL}\\ +\beta_{2}\;\mathrm{BMI}+\beta_{3}\;\mathrm{DM}+\beta_{4}\;\mathrm{age}+\beta_{5}\;\mathrm{sex} \end{array}$$

**FIGURE 2:**
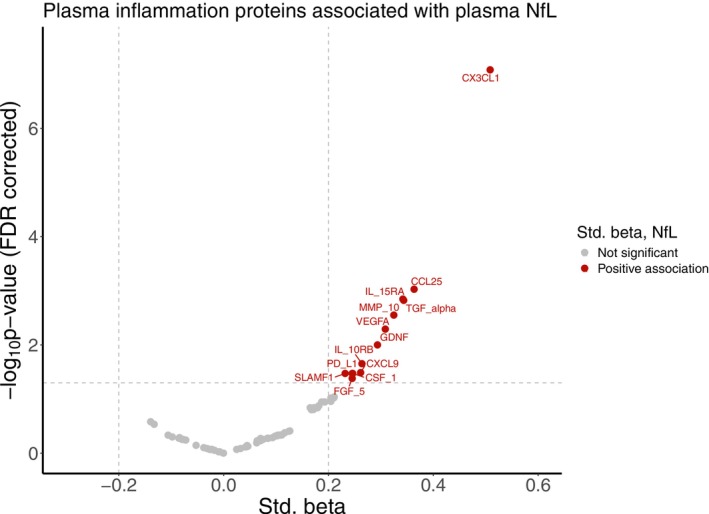
Volcano plot illustrating the positive association between plasma inflammatory markers and plasma NfL across the whole cohort.

In these analyses (Equation 4) we observed a positive association between NfL and inflammatory proteins that remained significant after multiple comparisons for the following 13 proteins: CCL25, CSF‐1, CX3CL1, CXCL9, FGF‐5, GDNF, IL‐10RB, IL‐15RA, MMP‐10, PD‐L1, SLAMF1, TGF‐alpha, VEGFA (all *q* < 0.05, corrected for multiple comparisons). The results of the statistical analyses (standardized β, *p*‐values and *q*‐values) are presented in Table 2, and the significant proteins (*q* < 0.05) are illustrated in a Volcano plot (Figure 2). The positive associations between plasma inflammation and NfL remained significant even after further adding entorhinal tau or middle/inferior temporal tau as independent predictors to the model, where tau was not a significant predictor in any of these models (data not shown).

**TABLE 2:** Statistical results showing a positive association of plasma inflammation markers (dependent variable) and plasma NfL, BMI, and DM (independent predictors).

Dependent variable	Independent predictors								
Inflammation marker	NfL			BMI			DM		
	std β	*p*	*q*	std β	*p*	*q*	std β	*p*	*q*
ADA	0.074	0.418	0.595	0.057	0.552	0.683	0.179	0.410	0.854
AXIN1	−0.023	0.799	0.910	0.093	0.331	0.522	−0.012	0.955	1.000
CASP‐8	0.109	0.230	0.436	0.175	0.067	0.220	−0.049	0.820	0.963
CCL11	0.190	0.030	0.110	−0.061	0.501	0.683	0.431	0.039	0.260
CCL19	0.019	0.821	0.911	0.240	0.007	0.110	0.595	0.004	0.066
CCL20	0.032	0.726	0.841	0.071	0.453	0.645	0.383	0.078	0.338
CCL23	0.088	0.339	0.553	−0.059	0.542	0.683	−0.064	0.769	0.943
CCL25	0.333	0.000	0.003	0.108	0.220	0.415	0.364	0.070	0.338
CCL28	0.172	0.046	0.163	−0.102	0.257	0.464	0.490	0.018	0.145
CCL3	0.005	0.953	0.997	0.060	0.537	0.683	0.003	0.989	1.000
CCL4	0.033	0.721	0.841	0.090	0.351	0.534	−0.041	0.853	0.986
CD244	0.101	0.256	0.441	0.023	0.806	0.852	0.382	0.073	0.338
CD40	0.119	0.181	0.400	0.241	0.011	0.120	0.084	0.694	0.936
CD5	0.228	0.010	0.052	0.168	0.068	0.220	0.075	0.719	0.936
CD6	0.002	0.979	0.997	0.176	0.061	0.220	0.099	0.639	0.928
CD8A	0.147	0.101	0.277	0.049	0.597	0.701	0.164	0.441	0.854
CDCP1	0.061	0.474	0.650	0.209	0.020	0.136	0.575	0.005	0.066
CSF‐1	0.222	0.011	0.052	0.290	0.002	0.029	0.145	0.480	0.889
CST5	0.206	0.020	0.081	0.124	0.176	0.382	0.166	0.426	0.854
CX3CL1	0.450	0.000	0.000	0.074	0.377	0.557	0.242	0.205	0.615
CXCL1	0.001	0.993	0.997	0.116	0.224	0.415	0.031	0.887	0.997
CXCL10	0.077	0.399	0.579	0.190	0.048	0.211	−0.198	0.364	0.854
CXCL11	0.123	0.184	0.400	0.058	0.547	0.683	−0.065	0.770	0.943
CXCL5	−0.059	0.519	0.681	0.065	0.495	0.683	0.128	0.554	0.924
CXCL6	0.000	0.997	0.997	0.042	0.651	0.742	0.537	0.013	0.119
CXCL9	0.244	0.007	0.051	0.100	0.286	0.480	−0.084	0.693	0.936
DNER	−0.058	0.525	0.681	−0.089	0.353	0.534	0.164	0.450	0.854
EN‐RAGE	0.081	0.378	0.569	0.019	0.840	0.852	−0.119	0.588	0.924
FGF‐5	0.268	0.003	0.026	0.021	0.831	0.852	−0.170	0.431	0.854
FGF‐19	0.062	0.487	0.655	−0.182	0.051	0.211	−0.121	0.566	0.924
FGF‐21	0.165	0.064	0.189	0.171	0.068	0.220	0.284	0.180	0.606
FGF‐23	0.224	0.012	0.055	0.124	0.181	0.383	0.339	0.108	0.422
Flt3L	−0.125	0.131	0.313	−0.133	0.125	0.327	0.781	0.000	0.009
GDNF	0.281	0.001	0.016	0.114	0.211	0.415	0.123	0.551	0.924
HGF	0.088	0.255	0.441	0.360	0.000	0.001	0.523	0.005	0.066
IFN‐gamma	0.142	0.117	0.309	0.170	0.074	0.230	−0.010	0.963	1.000
IL‐10RA	−0.108	0.236	0.437	−0.034	0.728	0.799	0.285	0.190	0.611
IL‐10RB	0.237	0.006	0.051	0.222	0.015	0.120	0.258	0.208	0.615
IL‐12B	0.230	0.008	0.052	0.222	0.014	0.120	0.237	0.244	0.668
IL‐15RA	0.371	0.000	0.000	0.129	0.128	0.327	−0.054	0.779	0.943
IL‐17A	0.088	0.344	0.553	−0.164	0.102	0.302	0.248	0.263	0.695
IL‐18R1	0.083	0.309	0.520	0.360	0.000	0.002	0.526	0.007	0.079
IL10	0.082	0.365	0.563	0.048	0.615	0.711	−0.093	0.665	0.936
IL18	0.113	0.208	0.429	0.091	0.330	0.522	0.365	0.088	0.361
IL6	0.082	0.355	0.559	0.310	0.001	0.025	0.000	1.000	1.000
IL7	−0.068	0.465	0.649	0.104	0.285	0.480	−0.027	0.902	0.997
IL8	0.100	0.256	0.441	−0.026	0.773	0.830	0.371	0.077	0.338
LAP‐TGF‐beta1	0.162	0.071	0.202	0.211	0.025	0.156	0.072	0.734	0.936
LIF‐R	0.165	0.051	0.171	0.030	0.735	0.799	0.729	0.000	0.009
MCP‐3	0.035	0.697	0.841	0.117	0.222	0.415	0.146	0.500	0.902
MCP‐1	0.077	0.385	0.569	0.019	0.840	0.852	0.395	0.065	0.338
MCP‐2	0.131	0.142	0.328	0.204	0.031	0.162	−0.116	0.584	0.924
MCP‐4	−0.019	0.825	0.911	0.234	0.012	0.120	0.114	0.586	0.924
MMP‐1	0.217	0.017	0.075	−0.053	0.572	0.683	0.074	0.729	0.936
MMP‐10	0.282	0.001	0.015	0.002	0.985	0.985	0.175	0.391	0.854
NT‐3	0.035	0.703	0.841	0.056	0.559	0.683	0.179	0.412	0.854
OPG	0.163	0.058	0.182	0.217	0.016	0.122	0.442	0.031	0.230
OSM	0.048	0.605	0.772	0.094	0.329	0.522	−0.027	0.903	0.997
PD‐L1	0.230	0.009	0.052	0.127	0.165	0.369	0.104	0.615	0.924
SCF	0.171	0.059	0.182	−0.140	0.141	0.347	−0.111	0.605	0.924
SIRT2	−0.011	0.906	0.974	0.106	0.276	0.480	−0.059	0.790	0.943
SLAMF1	0.212	0.011	0.052	0.050	0.565	0.683	0.726	0.000	0.009
ST1A1	−0.002	0.985	0.997	0.039	0.684	0.767	0.077	0.727	0.936
STAMBP	−0.032	0.727	0.841	0.074	0.444	0.645	−0.108	0.624	0.924
TGF‐alpha	0.301	0.001	0.011	0.125	0.164	0.369	0.184	0.366	0.854
TNF	0.141	0.123	0.313	0.124	0.194	0.399	−0.003	0.988	1.000
TNFB	−0.030	0.724	0.841	0.146	0.108	0.307	0.295	0.153	0.539
TNFRSF9	0.193	0.029	0.110	−0.052	0.572	0.683	−0.317	0.130	0.480
TNFSF14	−0.105	0.221	0.430	0.186	0.040	0.198	0.252	0.218	0.622
TRANCE	−0.010	0.908	0.974	0.150	0.114	0.312	0.000	1.000	1.000
TWEAK	−0.130	0.129	0.313	−0.197	0.029	0.162	0.192	0.345	0.854
uPA	0.109	0.215	0.429	0.131	0.156	0.369	0.165	0.432	0.854
VEGFA	0.287	0.001	0.015	0.165	0.066	0.220	0.382	0.062	0.338
4E‐BP1	0.114	0.213	0.429	0.187	0.051	0.211	−0.011	0.959	1.000

#### 3.3.4 Associations Between Performance in Episodic Memory, Tau PET, and Plasma Inflammation Across the Whole Study Sample

As a first step, we explored the associations between episodic memory performance and each of the individual demographics and clinical data. Higher years of education and being female were associated with better performance in both episodic memory tests, while higher age had a trend‐level negative association with episodic memory performance. Then, we checked the association of all imaging and plasma biomarkers with episodic memory performance using episodic memory as the dependent variable, and including age, sex, and years of education as covariates. Neither global Aβ PET nor regional tau PET burden were associated with episodic memory performance. WMH had a trend‐level negative association with SRT, delayed recall (std. β [95% CI] = −0.163 [−0.326 to −0.0003], *p* = 0.052). AD‐signature CTh had a trend‐level positive association with SRT, delayed recall (std. β [95% CI] = 0.145 [−0.020 to 0.310], *p* = 0.085). Plasma NfL was negatively associated with SRT, delayed recall (std. β [95% CI] = −0.220 [−0.375 to −0.065], *p* = 0.006). Compared to Group 0 (Aβ‐low, NfL‐low), Group 3 (Aβ‐low, NfL‐high) had lower SRT, immediate recall (std. β [95% CI] = −0.545 [−0.982 to −0.108], *p* = 0.016) and lower SRT, delayed recall (std. β [95% CI] = −0.668 [−1.103 to −0.233], *p* = 0.003), both models adjusted for age, sex, and education; the results for SRT, delayed recall are illustrated in Figure 1F.

We further performed an exploratory analysis using 13 selected plasma inflammatory proteins (which were previously found to be positively associated with plasma NfL) to investigate whether any of these proteins were associated with episodic memory in a model that included age, sex, and education as independent predictors. We found that three plasma inflammatory proteins (CCL25, CSF‐1 and VEGFA) were negatively associated with both immediate and delayed episodic memory performance (results for delayed episodic memory are presented on Table 3, *p* < 0.05, uncorrected for multiple comparisons), while higher education remained a strong significant predictor of better episodic memory performance in all the models. When adding plasma NfL as an independent predictor in the models described by Equation (4), only one of the plasma inflammatory markers (CCL25) remained negatively associated with immediate episodic memory (and not significantly for delayed episodic memory).

**TABLE 3:** Statistical results showing a negative association between episodic memory performance (SRT delayed recall, dependent variable) and plasma inflammatory proteins (independent predictors), and a positive association between SRT delayed recall and education.

Inflammation markers	std β.inflam	p.inflam	std β.educ	p.educ
CCL25	−0.180	0.029	0.310	0.000
CSF‐1	−0.175	0.032	0.303	0.000
CX3CL1	−0.130	0.114	0.317	0.000
CXCL9	−0.047	0.573	0.317	0.000
FGF‐5	−0.098	0.263	0.326	0.000
GDNF	−0.153	0.067	0.301	0.000
IL‐10RB	−0.081	0.330	0.310	0.000
IL‐15RA	−0.001	0.992	0.318	0.000
MMP‐10	−0.128	0.130	0.310	0.000
PD‐L1	−0.073	0.389	0.308	0.000
SLAMF1	0.028	0.749	0.314	0.000
TGF‐alpha	−0.036	0.671	0.315	0.000
VEGFA	−0.196	0.018	0.285	0.001

*Note:* All models included age and sex as confounding factors.

## 4 Discussion

**FIGURE 3:**
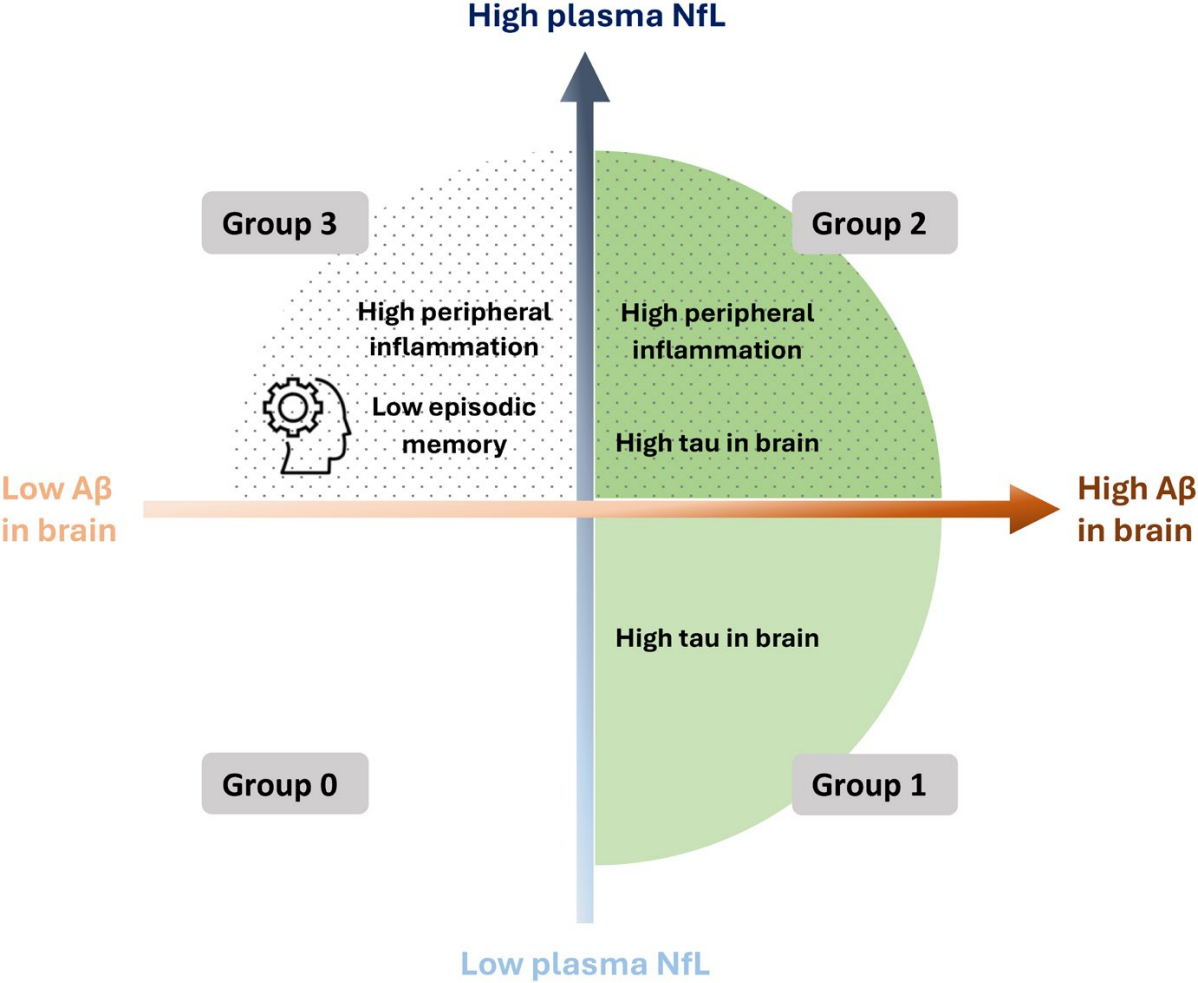
Schematic diagram of the overall study design. Groups were defined by median‐split dichotomization of global Aβ PET and plasma NfL levels: Group 0 = Aβ‐low/NfL‐low; Group 1 = Aβ‐high/NfL‐low; Group 2 = Aβ‐high/NfL‐high; Group 3 = Aβ‐low/NfL‐high.

In this study of 132 late middle‐aged Hispanic adults, we primarily focused on investigating the patterns of in vivo tau PET and peripheral inflammatory biomarkers in CU individuals across different stages defined by Aβ burden and neuronal injury. A schematic diagram of the overall study design is illustrated in Figure 3, and a summary of the statistical group comparisons in the study is presented in Table 4. Compared with Group 0 (Aβ‐low, NfL‐low), Group 1—which displayed an increase in Aβ without apparent neuronal damage—revealed a suggestive rise in middle/inferior temporal tau, indicating high sensitivity of ^18^F‐MK6240 to detect early neocortical tau pathology, even in the absence of evident neuronal injury. The absence of this trend in the entorhinal cortex could be attributed to the lower sensitivity of ^18^F‐MK6240 for the very initial stages of tau pathology in this region. In Group 2 (Aβ‐high, NfL‐high), ^18^F‐MK6240 detected tau in the middle/inferior temporal cortex at trend level and significantly detected entorhinal tau burden. Group 3 (Aβ‐low, NfL‐high) showed no uptake of ^18^F‐MK6240, indicating the absence of tau pathology. These observations resonate with established scientific literature wherein tau pathology is known to follow Aβ buildup, often preluding more significant neurodegeneration and cognitive decline (Hardy and Selkoe 2002). Even in Aβ‐negative older adults (Braak and Braak 1997; Maass et al. 2017), tau aggregates originating in the transentorhinal cortex tend to spread along functionally connected neuronal networks (Adams et al. 2019; Berron et al. 2021; Braak and Braak 1985, 1991; Kaufman et al. 2018). Further research is needed to elucidate the driving forces behind tau propagation and its potential impact on cognitive decline.

**TABLE 4:** Summary of statistical group comparisons in the study.

Group comparisons	^18^F‐MK6240 tau PET	Plasma inflammatory markers	AD‐signature cortical thickness (CTh)	White matter hyperintensities (WMH)	Episodic memory (SRT)
Group 1 vs. Group 0	High (#) in middle‐inferior temporal cortex	NS	NS	NS	NS
Group 2 vs. Group 0	High (***) in entorhinal cortex High (#) in middle‐inferior temporal cortex	High (*) in 8 markers (CCL25, CD244, CX3CL1, FGF‐19, IL‐15RA, IL8, MCP‐2, VEGFA)	NS	NS	NS
Group 3 vs. Group 0	NS	High (*) in 3 markers (CCL25, CX3CL1, GDNF)	Low (*)	High (#)	Low (*) in immediate recall Low (**) in delayed recall

*Note:* Groups were defined by median‐split dichotomization of global Aβ PET and plasma NfL levels: Group 0 = Aβ‐low/NfL‐low; Group 1 = Aβ‐high/NfL‐low; Group 2 = Aβ‐high/NfL‐high; Group 3 = Aβ‐low/NfL‐high. Abbreviations: Aβ, amyloid‐β; NfL, neurofilament light; NS, not significant; SRT, selective reminding test. Significant findings are indicated with symbols: *p < 0.05, **p < 0.01, ***p < 0.001. Statistical trend was denoted by ^#^ *p* < 0.1.

In a previous report on an aged population without dementia, elevated plasma NfL levels were associated with faster cognitive decline, suggesting that NfL may be a useful biomarker for tracking disease progression (Mielke et al. 2019). A recent study showed that in a CU aged population, CSF NfL levels were correlated with tau pathology and neurodegeneration (Arvidsson Rådestig et al. 2023). However, NfL is not a specific biomarker of AD, but rather a general indicator of ongoing neurodegeneration across several neurodegenerative and neurological disorders (Barro and Zetterberg 2021; Lin et al. 2018; Mattsson et al. 2017). Consistent with previous evidence, the group with low Aβ and high NfL (Group 3) did not show elevated tau burden but more severe cortical thinning and a trend toward CVI (as measured by high WMH), adding to the evidence that low Aβ and high neurodegeneration individuals (also known as suspected non‐Alzheimer pathology, or SNAP) are not on the AD pathway (Jack, Knopman, et al. 2016). This aligns with the interpretation that NfL reflects disease aggressiveness, rather than staging, and that combining Aβ and NfL levels can help characterize whether an individual is on the AD pathway and the disease aggressiveness (Jung and Damoiseaux 2024).

An emerging body of evidence suggests that glial cell activation represents a significant histopathological observation in AD brains (Heneka et al. 2015; Johnson et al. 2002) and that neuroinflammation might precede the onset of cognitive symptoms. Our study adds novel insights into the patterns of plasma inflammatory protein concentrations across stages defined by dichotomous Aβ and neuronal injury biomarkers. We found that Group 1 did not show elevated plasma inflammatory proteins compared to Group 0. In contrast, both Groups 2 and 3, characterized by high plasma NfL, showed higher levels of plasma inflammatory markers compared to Group 0. Our results suggest that peripheral inflammation is strongly linked to neuronal injury as measured by plasma NfL, regardless of whether Aβ is high (Group 2) or low (Group 3), consistent with evidence of inflammation being common across different neurological and neurodegenerative diseases (Ahmad et al. 2022).

Using general linear models across the whole cohort, we investigated the potential influence of risk factors and comorbidities on plasma inflammatory protein levels. In particular, we found that high peripheral inflammatory levels were associated with high NfL, high BMI, and DM, but not with specific markers of AD pathology (Aβ or tau). These elevated levels of inflammatory proteins previously linked to both aging and AD pathology participate in processes like cell adhesion, inflammatory response, cytokine response, chemotaxis, and others (Cullen et al. 2021). They also mirror those observed in DM (Ponce‐de‐Leon et al. 2022), underscoring the challenge of distinguishing between age‐related changes, comorbidity‐related alterations, and AD‐specific inflammation. From our findings, one might posit that multiple potential underlying mechanisms contribute to neuronal injury and possibly future cognitive decline, including tau burden with accumulating Aβ levels and separate inflammation pathways associated with high NfL, BMI, and DM.

Thus, in synthesizing our results with established literature, we propose three potential pathological pathways: (1) an AD pathology‐specific route, where abnormal Aβ and tau depositions may eventually lead to inflammation and neuronal injury (Ismail et al. 2020); (2) a comorbidity‐driven pathway, where comorbidities primarily initiate chronic inflammation, subsequently leading to neuronal injury, possibly enhancing the risk of AD‐like pathology (Balasubramanian et al. 2021); (3) a parallel contribution pathway, where inflammation and AD pathology coexist, independently contributing to cognitive deterioration. This multi‐pathway hypothesis implies that certain inflammatory markers might flag CU individuals who will later develop dementia.

Given that subtle cognitive decline might start in late middle age, we also explored which biomarkers predicted lower episodic memory performance in this cohort and found that the classical AD biomarkers, Aβ and tau, did not significantly associate with lower episodic memory. Instead, low episodic memory was linked to markers of neuronal injury (high NfL, low CTh), CVI (high WMH), and selected plasma inflammatory markers. Our findings align with studies advocating investigation into alternative biological mechanisms, beyond Aβ and tau, in aging and preclinical AD, which show apparent changes linked to subtle disturbances in episodic memory, even in CU late middle‐aged individuals (Malek‐Ahmadi et al. 2023).

A recent multi‐center study highlighted the heterogeneity within ATN groups: while some A+T+ individuals remained resilient, a small percentage of A−T− progressed to MCI (Ossenkoppele et al. 2022), emphasizing the need for further research into resilience and risk factors.

Our study also added insights into the influence of age, sex, and APOE4 on different biomarkers. First, we found that AD‐specific markers Aβ and tau were promoted by APOE4 carrier status and being female, but age played no role. Our findings support prior research showing an increased susceptibility to AD in female APOE4 carriers (Lin et al. 2015; Zokaei et al. 2017). Moreover, while the influence of the APOE4 allele is generally considered subtle in middle‐aged CU individuals (possibly attenuated by ongoing compensatory mechanisms) (Cacciaglia et al. 2019) our study identified its effects in this Hispanic population. Recent literature has shown that the magnitude of the APOE4 effect differs across Hispanic subgroups (Granot‐Hershkovitz et al. 2023), with some populations (e.g., Mexican Americans) showing weaker or non‐significant associations with AD or related dementias, while others (e.g., Central or South American ancestry) retain a stronger risk effect (Huggins et al. 2023). These differences are likely driven by genetic “admixture” (varying proportions of Amerindian, African, and European ancestry), as well as environmental and sociocultural factors (Naslavsky et al. 2022). Importantly, while prior work has focused primarily on clinical diagnosis or cognitive outcomes, PET‐based biomarker studies of Aβ and especially tau pathology in Hispanic cohorts remain scarce. Our study adds value by documenting these effects in a midlife Hispanic cohort, demonstrating that APOE4 and sex effects on biomarker burden are also observable, supporting the generalizability of these biological mechanisms while underscoring the need for further ancestry‐stratified analyses. In contrast, neither plasma inflammation, plasma NfL, CTh, nor WMH were influenced by sex or APOE4 status, but older age was associated with greater cortical thinning and more severe CVI. These results add support to the concept that there are independent neuropathological mechanisms in older adults. While Aβ and tau accumulation were influenced by genetics and sex, peripheral inflammation was associated with neuronal injury and comorbidities such as high BMI and DM, rather than APOE4 or sex. With aging, factors such as inflammation and CVI could accumulate and be important mechanisms contributing over time to exacerbate Aβ and tau pathology in the brain, worsening cognitive performance and leading to faster cognitive decline, but they may also independently worsen cognitive performance, irrespective of Aβ and tau pathologies.

The primary strengths of this study lie in its multimodal approach, encompassing a broad range of biomarkers, and its focus on the Hispanic population, a crucially underserved demographic group in research, highlighting the need to understand biological variabilities across ethnic groups. Additionally, by examining AD biomarkers in CU individuals, our study offers insights into potential AD preclinical stages and inter‐individual heterogeneity.

The study has some limitations. First, while the sample size was adequate, a larger sample would have strengthened the generalizability of our conclusions. Second, the study targeted late middle‐aged Hispanic adults, potentially restricting its generalizability to broader age and more diverse ethnic groups. A critical note for the interpretation of our results is that a high Aβ level as defined by the median split (SUVR = 1.125) does not necessarily indicate an Aβ‐positive status (A+) as defined by previously published cut‐offs (SUVR = 1.34) (Rippon et al. 2022), and similarly, elevated levels of NfL as defined by the median split (10.85 pg/mL) do not directly translate to a positive NfL status as previously published (20 pg/mL in the age range of 61–70 years old) (Simrén et al. 2022). A more comprehensive analysis incorporating a wider range of comorbidities and clinical history, such as a history of depression, could enhance the understanding of the results. Diversifying cognitive assessments focused on different cognitive domains would also allow for the exploration of potential correlations between specific markers and various patterns of cognitive decline.

In conclusion, the strong association between elevated peripheral inflammation and NfL levels underscored the significance of inflammation in neuronal damage, independent of Aβ and tau status in this cohort. The sensitivity of ^18^F‐MK6240 in detecting early neocortical tau pathology, even without apparent neuronal injury, provides valuable insights into the progression of AD‐like pathology. Future research requires a longitudinal approach with diverse cohorts to validate these findings and disentangle the complex interplay of AD‐specific and non‐specific pathologies, ultimately contributing to the development of more tailored intervention strategies from early AD preclinical stages.

## Author Contributions

**Mona‐Lisa Malarte:** conceptualization, methodology, writing – original draft, writing – review and editing, formal analysis, investigation, data curation, project administration, visualization. **Konstantinos Chiotis:** methodology, writing – review and editing, investigation. **Konstantinos Ioannou:** investigation, methodology, writing – review and editing. **Elena Rodriguez‐Vieitez:** investigation, methodology, writing – original draft, writing – review and editing, supervision, formal analysis, funding acquisition, conceptualization, project administration, data curation, visualization.

## Conflicts of Interest

The authors declare no conflicts of interest.

## Data Availability

All data used in this study was obtained from the Interdisciplinary Research to Understand the Interplay of Diabetes and Alzheimer's Disease (DiCAD) study at Columbia University. These data are publicly available and can be accessed upon request from the AD Knowledge Portal, managed by the US National Institute of Aging's Alzheimer's Disease Translational Research Program, https://adknowledgeportal.synapse.org, under a signed Controlled‐Access Data Use Certificate to comply with ethical guidelines for protection of human subjects.
